# Genomic epidemiology and temperature dependency of hypermucoviscous *Klebsiella pneumoniae* in Japan

**DOI:** 10.1099/mgen.0.000827

**Published:** 2022-05-27

**Authors:** Mi Nguyen-Tra Le, Shizuo Kayama, Kelly L. Wyres, Liansheng Yu, Junzo Hisatsune, Masato Suzuki, Koji Yahara, Tsuneko Terachi, Kana Sawa, Shin Takahashi, Toshihiko Okuhara, Kunihiko Kohama, Kathryn E. Holt, Tetsu Mizutani, Hiroki Ohge, Motoyuki Sugai

**Affiliations:** ^1^​ Department of Antimicrobial Resistance, Hiroshima University Graduate School of Biomedical and Health Sciences, Hiroshima, Japan; ^2^​ Project Research Center for Nosocomial Infectious Diseases, Hiroshima University, Hiroshima, Japan; ^3^​ Department of Bacteriology, Hiroshima University Graduate School of Biomedical and Health Sciences, Hiroshima, Japan; ^4^​ Antimicrobial Resistance Research Center, National Institute of Infectious Diseases, Japan; ^5^​ Department of Infectious Diseases, Monash University, Melboune, Victoria, Australia; ^6^​ Department of Clinical Laboratory, Osaka Police Hospital, Osaka, Japan; ^7^​ Fukuyama Rinsho (FML), Hiroshima, Japan; ^8^​ Department of Clinical Laboratory, Chugoku Rosai Hospital, Hiroshima, Japan; ^9^​ London School of Hygiene and Tropical Medicine, London, UK; ^10^​ Department of Infectious Diseases, Hiroshima University Hospital, Hiroshima, Japan

**Keywords:** *Klebsiella pneumoniae*, hypermucoviscous, hypervirulent, multidrug resistance, string test

## Abstract

*
Klebsiella pneumoniae* (Kp) has emerged as a global life-threatening pathogen owing to its multidrug resistance and hypervirulence phenotype. Several fatal outbreaks of carbapenem-resistant hypervirulent Kp have been reported recently. Hypermucoviscosity (HMV) is a phenotype commonly associated with hypervirulence of Kp, which is usually regulated by *rmpA* or *rmpA2* (regulators of the mucoid phenotype). Here, we found that temperature was important in the HMV phenotype of Kp, and the impact of temperature on HMV was not uniform among strains. We investigated the HMV phenotype at 37 °C and room temperature (20–25 °C) in 170 clinically isolated hypermucoviscous Kp strains in Japan and analysed the association between the HMV phenotype, virulence genes and antimicrobial resistance (AMR) genes. String length distribution at different temperatures was correlated with the genomic population of Kp. The strains carrying *rmpA*/*rmpA2* frequently showed the HMV phenotype at 37 °C, while the strains negative for these genes tended to show the HMV phenotype at room temperature. Hypervirulent Kp clusters carrying *rmpA*/*rmpA2* without extended-spectrum beta-lactamases (ESBL)/carbapenemases produced higher string lengths at 37 °C than at room temperature, and were mostly isolated from the respiratory tract. Other HMV strains showed distinct characteristics of not carrying *rmpA*/*rmpA2* but were positive for ESBL/carbapenemases, with a higher string length at room temperature than at 37 °C, and were frequently isolated from bloodstream infections. In total, 21 (13.5 %) HMV isolates carried ESBL and carbapenemases, among which five isolates were carbapenem-resistant hypervirulent Kp with a pLVPK-like plasmid (an epidemic virulence plasmid) and a pKPI-6-like plasmid (an epidemic *bla*_IMP-6_-bearing plasmid in Japan), suggesting the convergence of worldwide hypervirulence and epidemic AMR in Japan.

## Data Summary

The authors confirm that all supporting data, code and protocols have been provided within the article or through supplementary data files.

**Impact Statement**

Hypermucoviscosity is considered a phenotype usually associated with hypervirulence of the pathogen *Klebsiella pneumoniae *; however, the effect of temperature on hypermucoviscosity has not been studied. We investigated the hypermucoviscosity phenotype at both 37 °C and room temperature (20–25 °C) and measured the length of the strings. We discovered temperature-dependent differences in string length patterns, with the hypervirulent clones showing higher hypermucoviscosity at 37 °C, whereas the other clones, including the isolates with antimicrobial resistance, showed higher hypermucoviscosity at room temperature. These findings will facilitate the discovery of unknown factor(s) responsible for hypermucoviscosity at room temperature and may explain the different virulence tactics of *K. pneumoniae* infection.

## Introduction

*Klebsiella pneumoniae* (Kp) is currently regarded as a significant threat to global health because of the emergence of hypervirulent (hv) clones causing severe community-acquired infections and multidrug-resistant (MDR) clones related to hospital outbreaks [1]. Kp infections can be community- or hospital-acquired, leading to serious diseases such as pneumonia, primary pyogenic liver abscess or distinctive invasive syndrome. The virulence and resistance determinants are distributed in distinct subpopulations of Kp [1]; however, the emergence of Kp with both carbapenem-resistant and hv phenotypes was recently reported in China [2], South and South-East Asian countries [3], and various other regions globally [4]. The geographical focus of this convergence is likely to occur in Asia because of the common existence of both hv and MDR clones. As Japan is a part of Asia, hv/MDR Kp might have been disseminated into the main continent and evolved. In West Japan, carbapenemase-producing Kp has emerged in the past decade; these strains carry extended-spectrum β-lactamases (ESBL) (*bla*_CTX-M-2_) and carbapenemase (*bla*_IMP-6_) and are resistant to all β-lactam antibiotics, except imipenem [5]. Nevertheless, no studies have provided a complete molecular epidemiological investigation of the Kp population in Japan so far, leaving a gap in knowledge about the specific regional population and diversity.

A variety of Kp virulence factors have been identified, including capsular polysaccharide, colibactin, ferric ion uptake, salmochelin (*iro*) and aerobactin (*iuc*) [6]. hvKp strains display an increased ability to acquire iron than classical Kp (cKp) strains due to the synthesis of iron-acquisition factors, such as aerobactin or salmochelin, that ‘steal’ the iron from the host [7]. Another factor reported to contribute to the virulence of Kp is the hypermucoviscosity (HMV) phenotype [6–8]. HMV has traditionally been attributed to overexpression of the capsule, which assists these bacteria in colonizing the mucosa and protects them from phagocytosis and human defensin-mediated bactericidal activity [6]. The HMV phenotype is sometimes associated with the hypervirulence of Kp [8], although not all Kp with the HMV phenotype are hvKp [9]. The *magA* gene (mucoviscosity-associated gene A) was first identified to code for a factor responsible for HMV [10], but was later found to be specific to the capsular serotype K1 [11]. Nevertheless, strains other than the K1 serotype (*magA-*negative) have also been reported to have HMV and hv phenotypes [12], suggesting that another factor may be associated with the HMV phenotype besides *magA*. Recent studies have suggested that unknown factors other than capsule production also play a role in the HMV phenotype. The KpnO porin, an outer membrane protein, was found to contribute to capsular polysaccharide production in the hvKp NTUH-K2044 [13]. However, it remains unclear whether there is any difference in the distribution of this factor between hvKp and cKp. The HMV phenotype is reportedly enhanced by expression of the plasmid-borne loci *rmpADC* [14] (where *rmpA* is the regulator of the mucoid phenotype A) [7, 15–17] or *rmpA2* [7, 9, 17, 18], which are considered some of the major factors involved in the HMV phenotype [6, 7, 19]. Nevertheless, the relationship between the *rmpA* genes and the HMV phenotype remains unclear, as some HMV-positive isolates do not harbour these genes [9, 20]. In this context, the regulatory mechanism of HMV in Kp is not fully understood.

From the viewpoint of clinical practice, an effective diagnostic tool to predict hvKp and its related characteristics could provide valuable early warnings about the hypervirulence and potential metastatic infections; therefore, comprehensive knowledge of the genomic population, virulence determinants and resistance determinants may significantly contribute to the infection control strategy. Furthermore, elaborating our understanding of the regulatory mechanisms of HMV and virulence of Kp may advance the development and implementation of new chemotherapies targeting these factors, which would improve the prognosis of serious infections. From our preliminary data, we found that temperature was a key factor affecting the HMV of Kp. Here, we present a genomic epidemiological study of hypermucoviscous Kp that examines the genomic characteristics (sequence types, capsular types, *rmpA*/*rmpA2*, virulence genes and resistance genes) of hypermucoviscous Kp in correlation with the temperature-dependent HMV phenotype.

## Methods

### Bacterial isolates

A total of 236 Kp isolates, obtained from patient specimens, such as blood, respiratory tract (RT), urine, bile, pus or puncture fluid from different hospitals in the Kansai (southern-centre area of Japan) and Chugoku (middle-west area of Japan) regions between 2006 and 2017, were used for HMV evaluation by string tests. Strains were collected after completion of routine microbiological diagnostics. This study was approved by the ethical committees of the National Institute of Infectious Diseases Committee of Ethics (approval number 972).

### Modified string test

**Fig. 1.**
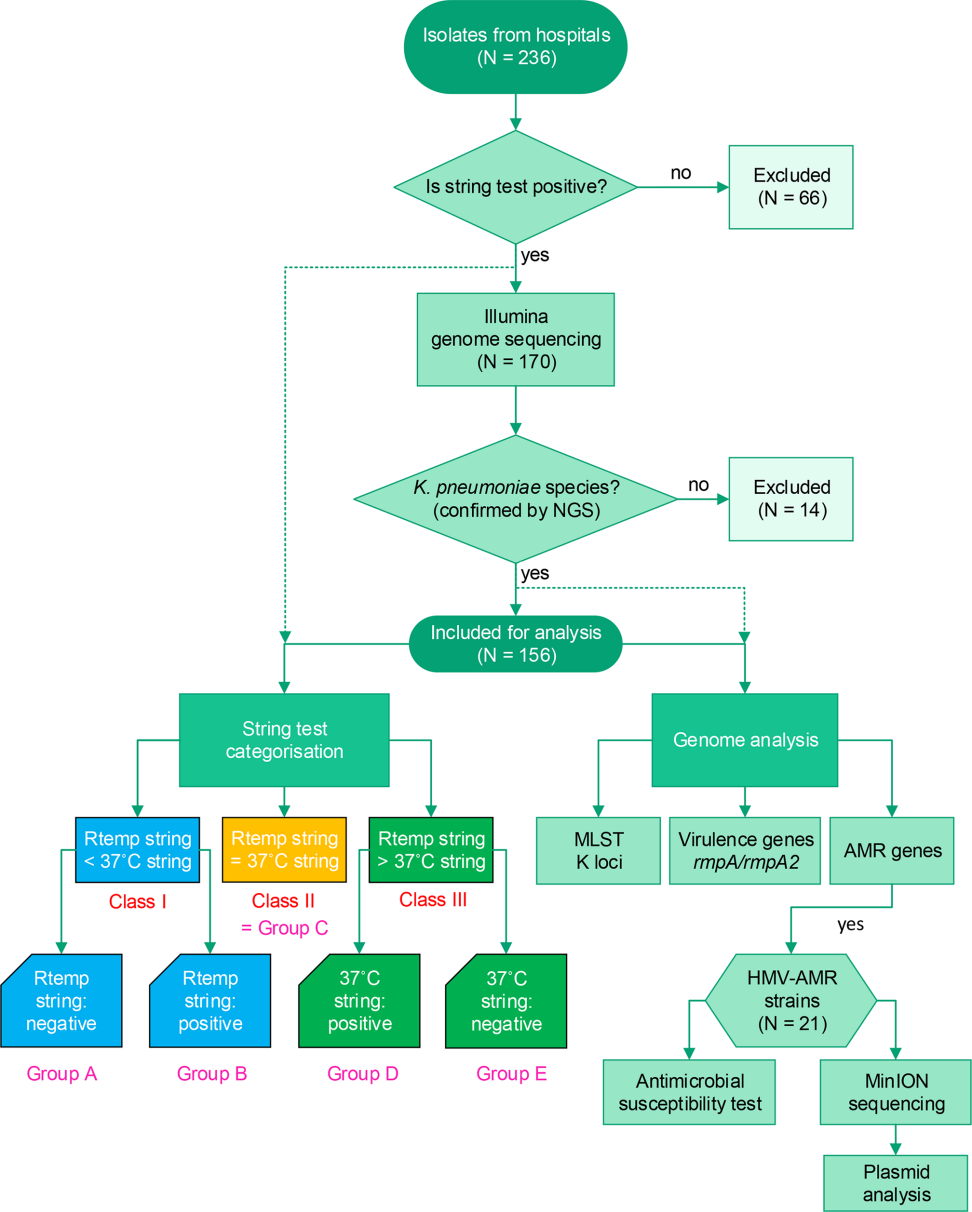
Flow chart of sample selection and data analysis processes.

The isolates were cultured on agar containing 5 % sheep blood (Becton, Dickinson) and incubated at 37 °C and room temperature (20–25 °C). Each blood agar plate was divided into four parts, and a full loop of each bacterial strain was streaked evenly in each quarter with a 2 mm inoculating loop. After 24, 48 and 72 h of incubation, a string test was performed with a cotton swab. A 5-mm-diameter cotton swab was used to collect all the colonies of a strain within the area and was stretched upward. The string test was deemed positive when a viscous string of ≥5 mm was generated. The string test at each time point was repeated five times, and the mean string length was recorded. The maximum string length at three time-points of each strain was used for data analysis. The strains showing negative string tests at all growth conditions were excluded, and 170 Kp isolates with a positive string test in at least one condition were included in subsequent investigations (Fig. 1).

**Table 1.** Comparison of key features between *K *. *pneumoniae* Class I and Class III

	Total (*n=*151)	Class I (*n=*109)	Class III (*n=*42)	OR (95 % CI)	*p-*value	
***Sequence type***						
ST23	27 (17.9 %)	26 (23.9 %)	1 (2.4 %)	12.843 (1.683–97.990)	0.002	**
ST65	19 (12.6 %)	18 (16.5 %)	1 (2.4 %)	8.110 (1.047–62.817)	0.019	*
ST86	14 (9.3 %)	14 (12.8 %)	0 (0.0 %)	n/a	0.011	*
ST268	6 (4.0 %)	6 (5.5 %)	0 (0.0 %)	n/a	0.187	
ST37	6 (4.0 %)	0 (0.0 %)	6 (14.3 %)	n/a	3.52×10^−4^	***
***K loci***						
KL1	29 (19.2 %)	28 (25.7 %)	1 (2.4 %)	14.173 (1.862–107.883)	0.001122	***
KL2	39 (25.8 %)	35 (32.1 %)	4 (9.5 %)	4.493 (1.487–13.579)	0.004	**
KL20	9 (6.0 %)	9 (8.3 %)	0 (0.0 %)	n/a	0.063	
KL57	10 (6.6 %)	10 (9.2 %)	0 (0.0 %)	n/a	0.062	
***Regulator of mucoid phenotype***						
intact *rmpA* (+)	95 (62.9 %)	92 (84.4 %)	3 (7.1 %)	70.353 (19.497–253.856)	1.285×10^−18^	***
intact *rmpA2* (+)	15 (9.9 %)	14 (12.8 %)	1 (2.4 %)	6.042 (0.769–47.480)	0.069	
intact *rmpA* and/or *rmpA2* (+)	97 (64.2 %)	93 (85.3 %)	4 (9.5 %)	55.219 (17.331–175.936)	3.104×10^−18^	***
***Virulence determinants***						
Yersiniabactin (*ybt*)	77 (51.0 %)	67 (61.5 %)	10 (23.8 %)	5.105 (2.275–11.453)	0.000034	***
Colibactin (*clb)*	39 (25.8 %)	37 (33.9 %)	2 (4.8 %)	10.278 (2.353–44.899)	0.000241	***
Aerobactin (*iuc*)	95 (62.9 %)	91 (83.5 %)	4 (9.5 %)	48.028 (15.243–151.324)	3.425×10^−17^	***
Salmochelin (*iro*)	101 (66.9 %)	97 (89.0 %)	4 (9.5 %)	76.792 (23.312–252.959)	1.437×10^−20^	***
Virulence score 0	37 (24.5 %)	7 (6.4 %)	30 (71.4 %)	0.027 (0.010–0.076)	8.645×10^−17^	***
Virulence score 1	19 (12.6 %)	11 (10.1 %)	8 (19.0 %)	0.477 (0.177–1.285)	0.137	
Virulence score 2	0 (0.0 %)	0 (0.0 %)	0 (0.0 %)	n/a	n/a	
Virulence score 3	37 (24.5 %)	35 (32.1 %)	2 (4.8 %)	9.459 (2.162–41.387)	4.63×10^−4^	***
Virulence score 4	19 (12.6 %)	19 (17.4 %)	0 (0.0 %)	n/a	0.002	**
Virulence score 5	39 (25.8 %)	37 (33.9 %)	2 (4.8 %)	10.278 (2.353–44.899)	2.41×10^−4^	***
***Antimicrobial resistance***						
ESBL	18 (11.9 %)	4 (3.7 %)	14 (33.3 %)	0.076 (0.023–0.250)	4.639×10^−7^	***
Carbapenemase	12 (7.9 %)	3 (2.8 %)	9 (21.4 %)	0.104 (0.027–0.406)	1.43×10^−4^	***
EBSL and Carbapenemase	11 (7.3 %)	3 (2.8 %)	8 (19.0 %)	0.120 (0.030–0.479)	5.55×10^−4^	**
***Temperature and string test***						
Mean string length at 37 °C (cm)		5.67 ± 0.63	4.04 ± 1.20		1.29×10^−4^	***
Mean string length at room temperature (cm)		0.10 ± 0.27	17.32 ± 3.91		<0.0001	***

Virulence score: 0, no acquired virulence loci detected; 1, only yersiniabactin (*ybt*); 2, only colibactin (*clb*) or colibactin and yersiniabactin; 3, only aerobactin (*iuc*) and/or salmochelin (*iro*) (without yersiniabactin or colibactin); 4, aerobactin and/or salmochelin with yersiniabactin (without colibactin); and 5, yersiniabactin, colibactin, aerobactin, and/or salmochelin.

Resistance score: 0, no ESBL and no carbapenemase, irrespective of colistin resistance; 1, only ESBL, but no carbapenemase, irrespective of colistin resistance; 2, carbapenemase without colistin resistance; and 3, carbapenemase with colistin resistance.*p < 0.05, **p < 0.01, ***p < 0.001.

**Table 2.** Comparison of key features between *K. pneumoniae* Class I group A and Class I group B

	Total (*n=*109)	Group A (*n=*83)	Group B (*n=*26)	OR (95 % CI)	*p-*value	
***Sequence type***						
ST23	26 (23.9 %)	23 (27.7 %)	3 (11.5 %)	2.939 (0.804–10.736)	0.091	
ST65	18 (16.5 %)	16 (19.3 %)	2 (7.7 %)	2.866 (0.613–13.396)	0.165	
ST86	14 (12.8 %)	6 (7.2 %)	8 (30.8 %)	0.175 (0.054–0.568)	3.738×10^−3^	**
ST268	6 (5.5 %)	6 (7.2 %)	0 (0.0 %)	n/a	0.332	
***K loci***						
KL1	28 (25.7 %)	25 (30.1 %)	3 (11.5 %)	3.305 (0.908–12.020)	0.058	
KL2	35 (32.1 %)	27 (32.5 %)	8 (30.8 %)	1.085 (0.419–2.808)	0.867	
KL20	9 (8.3 %)	8 (9.6 %)	1 (3.8 %)	2.667 (0.318–22.385)	0.683	
***Regulator of mucoid phenotype***						
intact *rmpA* (+)	92 (84.4 %)	77 (92.8 %)	15 (57.7 %)	9.411 (3.015–29.373)	9.9×10^−5^	***
intact *rmpA2* (+)	14 (12.8 %)	11 (13.3 %)	3 (11.1 %)	1.171 (0.301–4.564)	1.000	
intact *rmpA* and/or *rmpA2* (+)	93 (85.3 %)	78 (94.0 %)	15 (57.7 %)	11.440 (3.470–37.711)	4.1×10^−5^	***
***Virulence prediction***						
Yersiniabactin (*ybt*)	67 (61.5 %)	52 (62.7 %)	15 (57.7 %)	1.230 (0.502–3.014)	0.650	
Colibactin (*clb)*	37 (33.9 %)	34 (41.0 %)	3 (11.5 %)	5.320 (1.479–19.137)	0.006	**
Aerobactin (*iuc*)	91 (83.5 %)	75 (90.4 %)	16 (61.5 %)	5.859 (2.000–17.168)	5.52×10^−4^	***
Salmochelin (*iro*)	97 (89.0 %)	80 (96.4 %)	17 (65.4 %)	14.118 (3.488–57.686)	9.8×10^−5^	***
***Temperature and string test***						
Mean string length at 37 °C (cm)		3.93 ± 0.48	11.21 ± 1.74		2.898×10^−7^	***

*p< 0.05, **p< 0.01, ***p< 0.001.

**Table 3.** Comparison of key features of hypermucoviscous *K. pneumoniae* derived from the respiratory tract and blood specimens

	Total (*n=*101)	Respiratory tract (*n=*67)	Blood (*n=*34)	OR (95 % CI)	*p-*value	
***Sequence type***						
ST23	18 (17.8 %)	15 (22.4 %)	3 (8.8 %)	2.981 (0.799–11.124)	0.092	
ST65	14 (13.9 %)	13 (19.4 %)	1 (2.9 %)	7.944 (0.993–63.562)	0.031	*
ST86	10 (9.9 %)	10 (14.9 %)	0 (0.0 %)	n/a	0.015	*
ST268	5 (5.0 %)	4 (6.0 %)	1 (2.9 %)	2.095 (0.225–19.513)	0.661	
ST37	2 (2.0 %)	0 (0.0 %)	2 (5.9 %)	3.094 (2.326–4.114)	0.111	
***K loci***						
KL1	20 (19.8 %)	17 (25.4 %)	3 (8.8 %)	3.513 (0.951–12.977)	0.049	*
KL2	31 (30.7 %)	26 (38.8 %)	5 (14.7 %)	3.678 (1.263–10.709)	0.013	*
KL20	8 (7.9 %)	7 (10.4 %)	1 (2.9 %)	3.850 (0.454–32.655)	0.261	
***Regulator of mucoid phenotype***						
intact *rmpA* (+)	66 (65.3 %)	58 (86.6 %)	8 (23.5 %)	20.944 (7.265–60.379)	3.151×10^−10^	***
intact *rmpA2* (+)	8 (7.9 %)	6 (9.0 %)	2 (5.9 %)	1.574 (0.300–8.248)	0.714	
***Virulence prediction***						
Yersiniabactin (*ybt*)	52 (51.5 %)	39 (58.2 %)	13 (38.2 %)	2.250 (0.966–5.238)	0.058	
Colibactin (*clb)*	27 (26.7 %)	22 (32.8 %)	5 (14.7 %)	2.836 (0.965–8.328)	0.052	
Aerobactin (*iuc*)	67 (66.3 %)	59 (88.1 %)	8 (23.5 %)	23.969 (8.114–70.803)	8.861×10^−11^	***
Salmochelin (*iro*)	71 (70.3 %)	63 (94.0 %)	8 (23.5 %)	51.188 (14.172–184.885)	2.350×10^−13^	***
Virulence score 0	23 (22.8 %)	3 (4.5 %)	20 (58.8 %)	0.033 (0.009–0.126)	7.531×10^−10^	***
Virulence score 1	11 (10.9 %)	5 (7.5 %)	6 (17.6 %)	0.376 (0.106–1.337)	0.175	
Virulence score 2	0 (0.0 %)	0 (0.0 %)	0 (0.0 %)	n/a	n/a	
Virulence score 3	26 (25.7 %)	25 (37.3 %)	1 (2.9 %)	19.643 (2.528–152.602)	1.89×10^−4^	***
Virulence score 4	14 (13.9 %)	12 (17.9 %)	2 (5.9 %)	3.491 (0.734–16.597)	0.132	
Virulence score 5	27 (26.7 %)	22 (32.8 %)	5 (14.7 %)	2.836 (0.965–8.328)	0.052	
***Antimicrobial resistance***						
ESBL	4 (4.0 %)	0 (0.0 %)	4 (11.8 %)	n/a	0.011	*
Carbapenemase	0 (0.0 %)	0 (0.0 %)	0 (0.0 %)	n/a	n/a	
***Temperature and string test***						
Class I	74 (73.3 %)	62 (92.5 %)	12 (35.3 %)	22.733 (7.190–71.874)	8.110×10^−10^	***
Group A	59 (58.4 %)	52 (77.6 %)	7 (20.6 %)	13.371 (4.868–36.729)	3.914×10^−8^	***
Group B	15 (14.9 %)	10 (14.9 %)	5 (14.7 %)	1.018 (0.318–3.255)	0.977	
Class III	24 (23.8 %)	4 (6.0 %)	20 (58.8 %)	0.044 (0.013–0.151)	3.693×10^−9^	***
Group D	14 (13.9 %)	4 (6.0 %)	10 (29.4 %)	0.152 (0.044–0.533)	0.004	**
Group E	10 (9.9 %)	0 (0.0 %)	10 (29.4 %)	n/a	7×10^−6^	***
Class II (Group C)	3 (3.0 %)	1 (1.5 %)	2 (5.9 %)	0.242 (0.021–2.774)	0.261	
Mean string length at 37 °C (cm)		5.68 ± 0.91	3.63 ± 1.03		0.006	**
Mean string length at room temperature (cm)		1.08 ± 0.46	7.12 ± 2.12		1.995×10^−8^	**

Based on the string length at different temperatures, the isolates were separated into three classes: string length at room temperature was lower than that at 37 °C (Class I), string lengths at room temperature were equal to those at 37 °C (Class II) and string length at room temperature was higher than that at 37 °C (Class III). Class I was further divided into subgroups based on the pattern of string length: group A (positive string test at 37 °C but negative string test at room temperature) and group B (positive string test at both temperatures but higher string length at 37 °C). Similarly, Class III was subdivided into group D (positive string test at both temperatures but higher string length at room temperature) and group E (positive string test at room temperature but negative string test at 37 °C). Class II included only one group, namely group C (Fig. 1).

### Antimicrobial susceptibility testing

Antimicrobial susceptibility testing was performed using the broth microdilution method. The following drugs were tested: piperacillin (PIPC), cefazolin (CAZ), cefepime (CFPM), imipenem (IPM), meropenem (MEPM), doripenem (DRPM), aztreonam (AZT), ampicillin/sulbactam (ABPC/SBT), gentamycin (GM), tobramycin (TOB), amikacin (AMK), minocycline (MINO), levofloxacin (LVFX), ciprofloxacin (CPFX), sulfamethoxazole (ST), fosfomycin (FOM) and florfenicol (CP). The results of the minimum inhibitory concentrations (MICs) were interpreted based on the Clinical and Laboratory Standards Institute (CLSI) M100-S28 guidelines [21].

### DNA extraction, whole-genome sequencing and genomic analysis

All Kp isolates were cultured overnight in Luria-Bertani (LB) broth at 37 °C, followed by total DNA extraction using the phenol–chloroform method. Libraries were constructed using Nextera DNA kits (Illumina,) following the manufacturer’s instructions, and whole-genome sequencing (WGS) was performed using Illumina MiSeq, generating 150 bp paired-end reads. The samples in this study achieved a sequencing depth of coverage ranging from 30× to 110×. *De novo* assemblies were generated using Shovill v1.0.9 and subsequently annotated using the PATRIC RAST-tk-enabled Genome Annotation Service. The whole-genome sequences of our isolates were analysed using Kleborate v2.1.0 [22] with the Kaptive option [23] for their sequence types, K (capsule) serotype prediction, virulence loci and antimicrobial resistance (AMR) genes. The multi-locus sequence type (MLST) alleles (*gapA*, *infB*, *mdh*, *pgi*, *phoE*, *rpoB*, and *tonB*) and sequence type (ST) profiles that had not been previously described were submitted to the curator of the official Kp BIGSdb-Pasteur database (http://bigsdb.pasteur.fr/klebsiella/) to assign new designations. The virulence and resistance scores were automatically calculated using Kleborate based on the types of virulence and AMR genes carried by each isolate (https://github.com/katholt/Kleborate/wiki/Scores-and-counts). From the results of Kleborate, 14 isolates were identified as *K. quasipneumoniae* or *K. variicola* and hence were excluded from this study. The remaining 156 isolates were confirmed as *K. pneumoniae* and were used for further analysis (Fig. 1).

Whole-genome SNP analysis was performed with CSIPhylogeny 1.4 [24] from the Center for Genomic Epidemiology with default settings [minimum depth at SNP positions: 10, relative depth at SNP positions: 10, minimum distance between SNPs (prune): 10, minimum SNP quality: 30, minimum read mapping quality: 25, and minimum Z-score: 1.96]. A phylogenetic tree was created and was annotated with the Interactive Tree of Life (iTOL) [25]. Related nodes within the phylogenetic tree were clustered using RAMI [26]. With the threshold of 0.1, RAMI produced 48 clusters, among which eight predominant clusters were found, and their key features were compared.

Isolates carrying ESBL and/or carbapenemase genes were selected for long-read sequencing. Total genomic DNA was extracted using the Genomic DNA buffer set (Qiagen) and Qiagen Genomic-tip 20 G^−1^, according to the manufacturer’s instructions. The DNA library was prepared using the SQK-RBK004 Rapid Barcoding Kit, loaded onto a FLO-MIN106D R9.4.1 flow cell, and sequenced with MinION (Oxford Nanopore Technologies) for 72 h. Hybrid assembly of Illumina short reads and MinION long reads was performed using the hybrid assembler Unicycler v0.4.8 [27]. The complete sequences of virulence plasmids were selected and compared with the available virulence plasmids pLVPK (accession number AY378100), a well-characterized virulence plasmid in Kp, using blast (https://blast.ncbi.nlm.nih.gov/Blast.cgi), Blast Ring Image Generator (BRIG) v0.95 [28]. Additionally, the virulence plasmids from our ST36 Kp were compared with WCHKP13F2 (MF943217), an ST36Kp-derived virulence plasmid reported from China. The AMR plasmids were also extracted, and complete plasmid comparisons were performed. The *bla*_IMP-6_-carrying plasmids were compared with pKPI-6 (AB616660), a *bla*_IMP-6_-carrying epidemic plasmid in Japan. The plasmids encoding *bla*_KPC-2_ were compared with some publicly available *bla*_KPC-2_-harbouring plasmids such as pKPC-HvKP4 (MF437312), p675920-1 (MF133495), pHN7A8 (JN232517) and pKPC-LK30 (KC405622). The *bla*_CTX-M-15_-carrying plasmids were compared with two available plasmids encoding *bla*_CTX-M-15_, pAMA1416 (MG462728) and pCTX15_DHQP14000954 (CP016925).

### Statistical analysis

Statistical analysis was performed using IBM SPSS Statistics v28.0.1.0. Comparisons between either Class I and Class III isolates, group A and group B isolates, or RT and blood isolates were performed using the chi-square test and Fisher’s exact test (for key features) and Mann–Whitney test for mean string length. Mean string lengths at 37 °C and room temperature between the *rmpA/rmpA2* positive and negative groups were analysed using the Mann–Whitney test and Wilcoxon signed-rank test.

### Accession number(s)

The whole-genome sequence FASTQ files were deposited in DDBJ under BioProject nos. PRJDB12075, DRX309353–DRX309536 and DRX317549–DRX317564.

## Results

### Distribution of multi-locus STs and predicted capsular serotypes (K) within the HMV Kp population

**Fig. 2.**
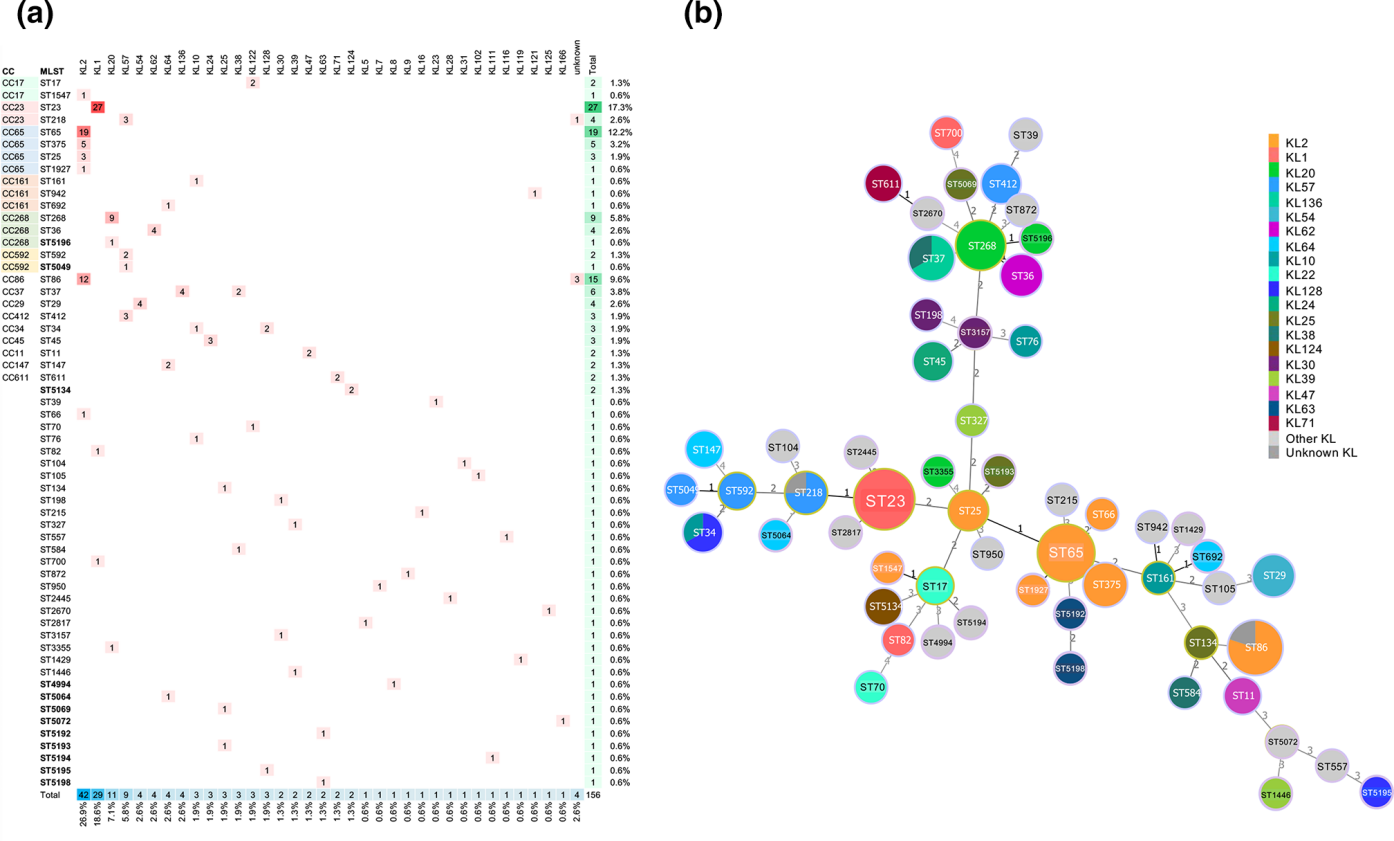
Sequence type (ST), clonal complex (CC) and capsular locus (KL) diversity among 156 hypermucoviscous *K. pneumoniae* isolates. (**a**) The number of genomes representing each KL by ST as well as the prevalence of each ST and KL are demonstrated. STs in bold indicate the newly identified STs in our study. Four strains having KL with low or no confidence as assessed by Kleborate and Kaptive are shown as ‘unknown KL’. Among these unidentified KL isolates, three belong to ST86 and only had 14/18 expected genes for KL2, and one belongs to ST218 and only carried 12/18 expected genes for KL57. (**b**) Minimum spanning tree showing the correlation between ST and KL present in our study.

Genotypic analysis using WGS on 156 HMV Kp strains showed that HMV Kp was highly diverse, comprising 58 STs and belonging to 35 known K-loci and four unknown K-loci (Fig. 2). Twelve (20.69%) of the 58 STs had not been previously identified (ST4994, ST5049, ST5064, ST5069, ST5072, ST5134, ST5192, ST5193, ST5194, ST5195, ST5196 and ST5198). The most prevalent STs were ST23 (*n=*27, 17.3%), ST65 (*n=*19, 12.2%), ST86 (*n=*15, 9.6%), ST268 (*n=*9, 5.8%), ST37 (*n=*6, 3.8%) and ST375 (*n=*5, 3.2%). The most prevalent K-loci, which accounted for >50 % of the isolates, were KL2 (*n=*42, 26.9%), KL1 (*n=*29, 18.6%), KL20 (*n=*11, 7.1%) and KL57 (*n=*9, 5.8%). While ST23, ST65 and ST268 were each associated with a single K-locus (KL1, KL2 and KL20, respectively), some other STs were associated with multiple K-loci; for example, ST37 included four KL136 and two KL38 (Fig. 2). Furthermore, 67.2 % (39/58) and 42.9 % (15/35) of the STs and known K-loci, respectively, were represented by a single isolate.

### AMR determinants and phenotypes

**Fig. 3.**
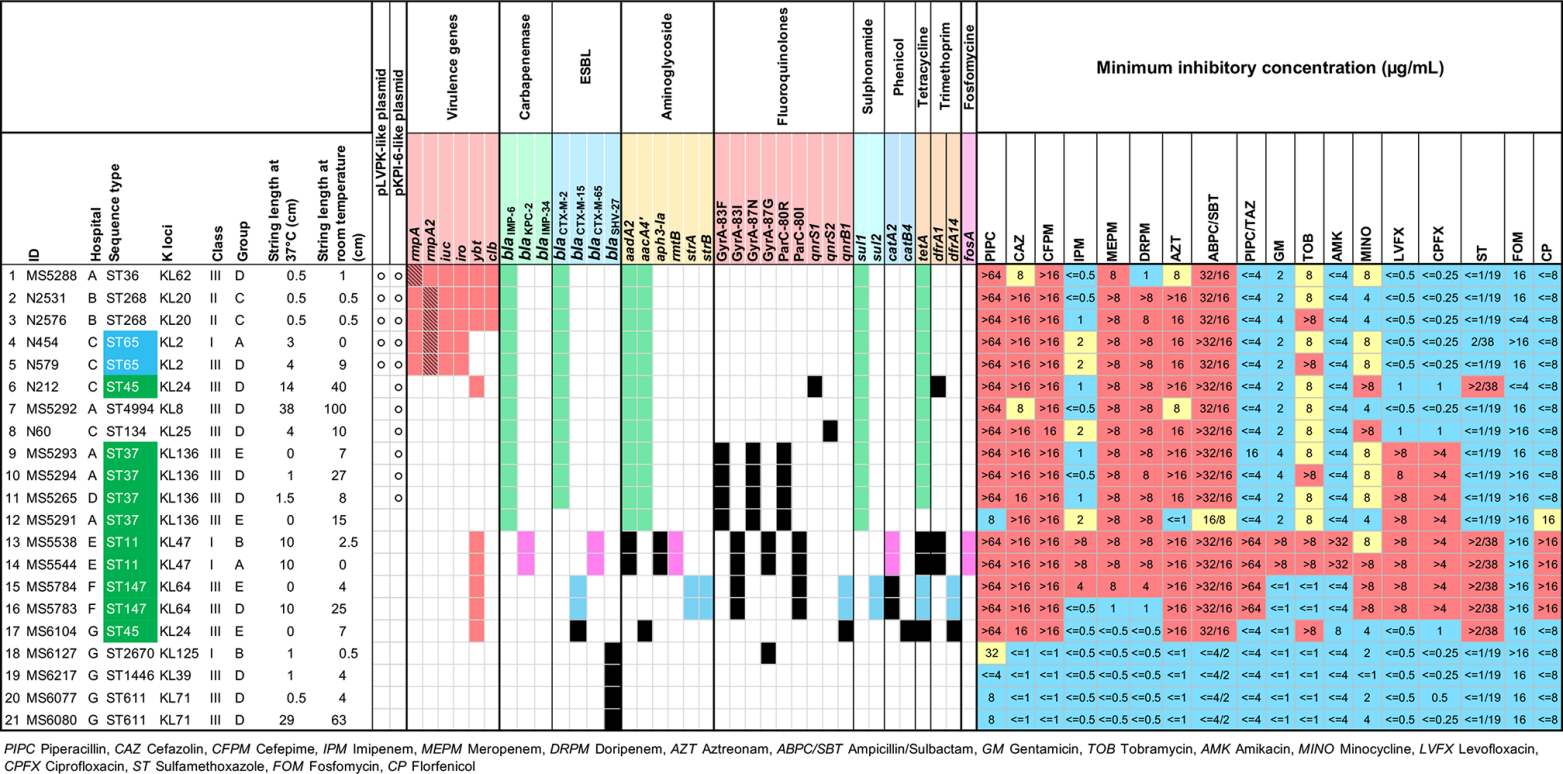
Key features of AMR-HMV *K. pneumoniae* isolates. Characteristics of 21 HMV-AMR isolates that carried ESBL/carbapenemase genes are demonstrated. The isolates from hypervirulence-background sequence types (STs) and AMR-background STs are highlighted in blue and green in the sequence type column, respectively. The presence of pLVPK-like and pKPI-6-like plasmids is marked with circles. The presence of intact or truncated *rmpA* and *rmpA2* genes is displayed as plain red colour or striped red colour, respectively. For the AMR genes, the same colours, except black, indicate that genes belonged to the same plasmid: green, pKPI-6-like plasmid; pink, *bla*_KPC-2_-carrying plasmid; blue, *bla*_CTX-M-15_-carrying plasmid; black indicates that genes belonged to the other plasmids or chromosome. Minimum inhibitory concentrations (µg ml^–1^) are interpreted as susceptible (blue), intermediate (yellow) or resistant (red).

In this collection, we detected 21 isolates (13.5%) that carried ESBL and/or carbapenemase genes. Among them, 13 isolates carried both ESBL and carbapenemase genes, seven isolates carried only ESBL, and two isolates carried only carbapenemases. The ESBL genes included *bla*_CTX-M-2_ (*n=*11, 52.4%), *bla*_SHV-27_ (*n=*4, 19.0%), *bla*_CTX-M-15_ (*n=*3, 14.3%) and *bla*_CTX-M-65_ (*n=*2, 9.5%), and the carbapenemase genes included *bla*_IMP-6_ (*n=*12, 57.1%) and *bla*_KPC-2_ (*n=*2, 9.5%) (Fig. 3).

Half of the ESBL- and/or carbapenemase gene-carrying isolates (*n=*11/21) carried both *bla*_CTX-M-2_ and *bla*_IMP-6_, along with *aacA4*, *aadA2*, *sul1* and *tetA*, all of which showed resistance to cephalosporins, meropenem and doripenem, as well as tobramycin and minocycline. Furthermore, four of these isolates (MS5293, MS5294, MS5265 and MS5291) also carried mutations associated with fluoroquinolone resistance (GyrA-83F, GyrA-87N and ParC-80R), resulting in resistance to levofloxacin and ciprofloxacin.

Two isolates (MS5538 and MS5544) possessed *bla*_KPC-2_, *bla*_CTX-M-65_ and *bla*_TEM-1B_, together with *aadA2*, *aph3-Ia*, *rmtB* (aminoglycoside resistance), *sul2* (sulfonamide resistance) and *catA2* (phenicol resistance). These also displayed extended resistance to all tested cephalosporins, carbapenems, aminoglycosides, sulfamethoxazole and florfenicol. Additionally, these two isolates carried chromosomal mutations (GyrA-83I, GyrA-87G and ParC-80I) consistent with the observed fluoroquinolone resistance phenotype.

### Genotypic convergence of AMR and HMV

**Fig. 4.**
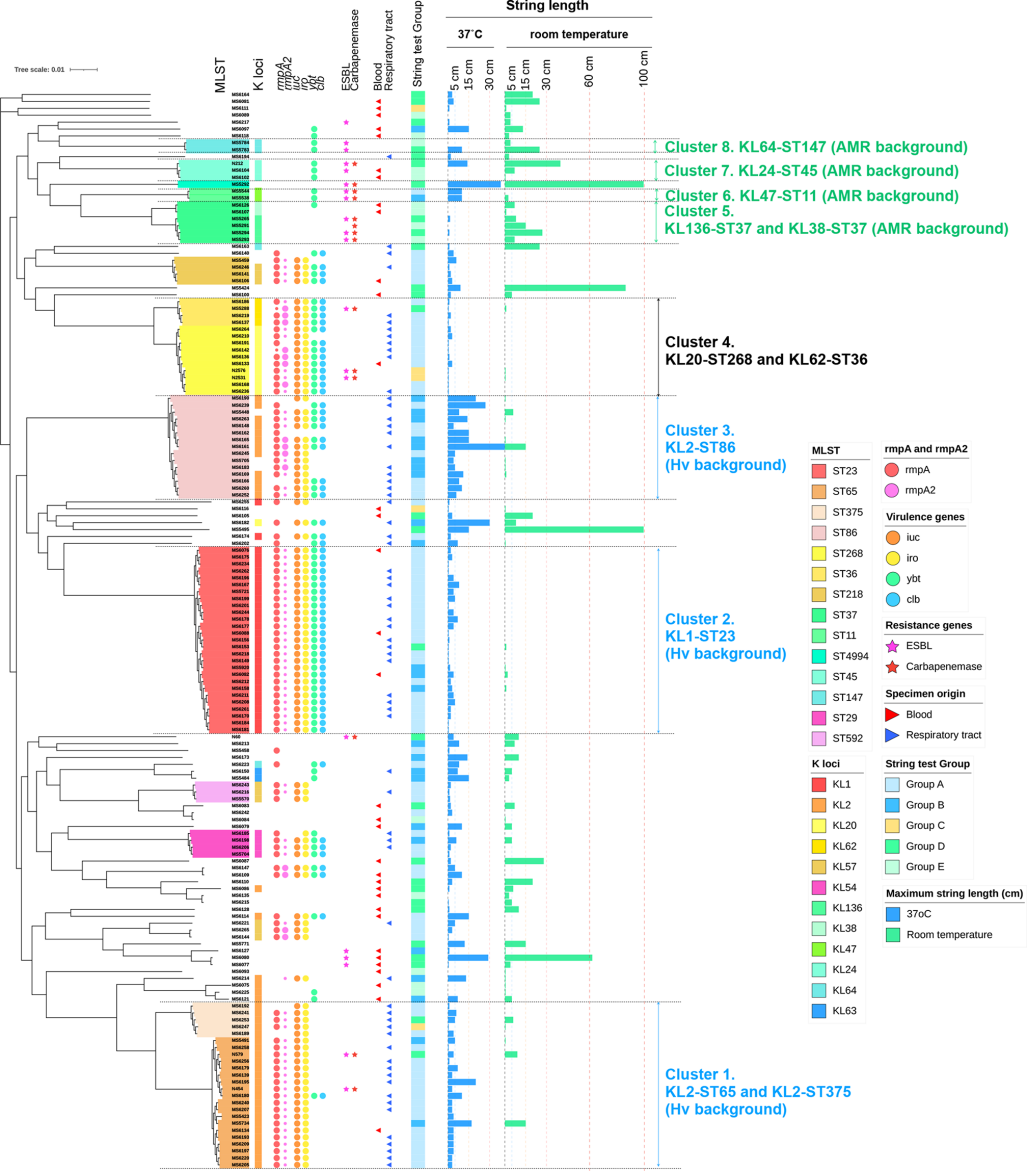
Phylogenetic analysis of 156 HMV *K. pneumoniae* isolates. Isolates are annotated with the datasets of sequence types (ST), capsular locus (KL), virulence genes, resistance genes, specimen origins, string test group, and maximal string length at 37 °C and room temperature, from left to right. Different STs are highlighted in different colours, and the most prominent clusters are marked as follows: cluster 1, KL2-ST65 and KL2-ST375; cluster 2, KL1-ST23; cluster 3, KL2-ST86; cluster 4, KL20-ST268 and KL62-ST36; cluster 5, KL136-ST37 and KL38-ST37; cluster 6, KL47-ST11; cluster 7, KL24-ST45; and cluster 8, KL64-ST147. For the *rmpA* and *rmpA2* genes, the large circle indicates an intact gene, while the small circle indicates a truncated gene.

Phylogenetic analysis (Fig. 4) revealed dominant clusters corresponding to the dominant STs described above and represented well-known hvKp and cKp clones: hv-KL2-ST65 and -KL2-ST375 (cluster 1), -KL1-ST23 (cluster 2) and -KL2-ST86 (cluster 3) possessed *rmpA*/*rmpA2* and other virulence determinants (*ybt, clb, iuc* and *iro*), but only a few carried ESBL/carbapenemases. Additionally, all KL2-ST65 and KL1-ST23 isolates carried an intact *rmpA* and a truncated *rmpA2*, while the other clusters did not show a consensus pattern. In contrast, the other clusters, including KL136-ST37 and KL38-ST3, KL47-ST11, KL24-ST45, and KL64-ST147 (clusters 5, 6, 7 and 8, respectively), mostly lacked *rmpA*/*rmpA2* and all other acquired virulence loci except *ybt,* but had a high prevalence of ESBL and/or carbapenemases (10/13 strains, 76.9%). Another cluster comprising KL20-ST268 and KL62-ST36 (cluster 4) displayed characteristics similar to those of cluster 2 (KL1-ST23), with a high prevalence of *rmpA*/*rmpA2* and other virulence determinants. These findings were generally consistent with the traditional view of Kp, for which acquired virulence and acquired resistance genes are usually found in distinct subsets of the population. However, we also identified several so-called ‘convergent’ isolates harbouring both acquired virulence and acquired AMR genes.

The 21 AMR-HMV isolates belonged to 14 different STs and 13 corresponding K-loci (Fig. 3). These STs included AMR-background STs [4] such as ST37 (*n=*4, 19.0%), ST11 (*n=*2, 9.5%), ST45 (*n=*2, 9.5%) and ST147 (*n=*2, 9.5%) (coloured in green), the hypervirulent-background STs [1, 4] such as ST65 (*n=*2, 9.5%) (coloured in blue), and some other miscellaneous STs such as ST268, ST36, ST134 and ST611.

**Fig. 5.**
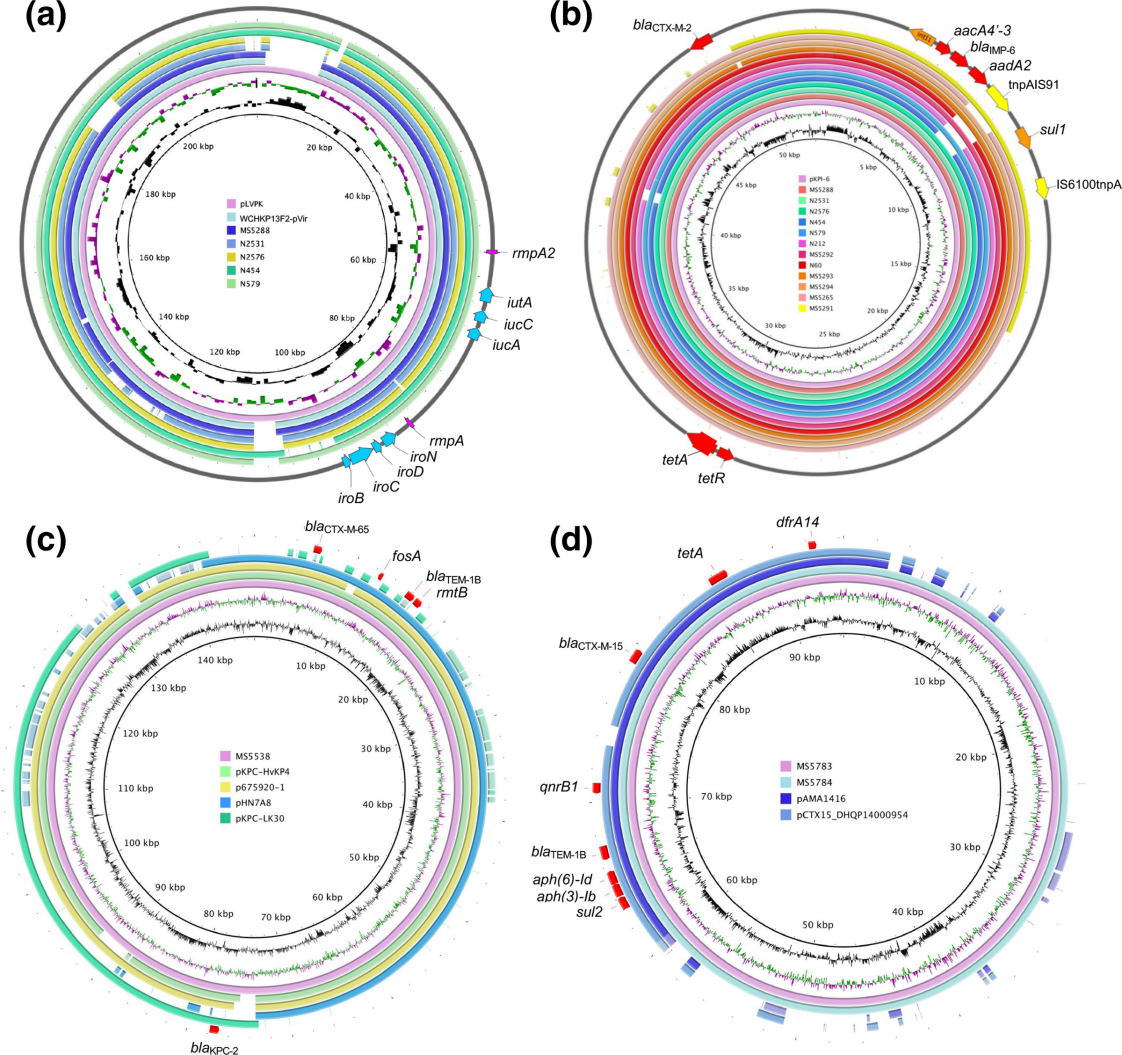
Plasmid comparisons of (a) *rmpA*/*rmpA2-*carrying plasmids, (**b**) *bla*_IMP-6_-carrying plasmids, (**c**) *bla*_KPC-2_ *, bla*_CTX-M-65_ and *bla*_TEM-1B_-carrying plasmids, and (d) *bla*_CTX-M-15_-carrying plasmids. (**a**) Structural comparison of the plasmid pLVPK, WCHKP13F2-pVir, and the *rmpA*/*rmpA2-*harbouring plasmids from MS5288, N2531, N2576, N454 and N579 strains using complete plasmid sequences. pLVPK was used as the reference and is shown as the innermost ring. The outermost circle displays the locations of *rmpA*, *rmpA2* and other virulence-coding genes (*iro, iuc* and *iut*) in pLVPK. (**b**) Structural comparison of the plasmid pKPI-6 and the *bla*_IMP-6_-carrying plasmids from MS5288, N2531, N2576, N454, N579, MS5292, N60, MS5293, MS5294, MS5265 and MS5291 strains using complete plasmid sequences. pKPI-6 was used as the reference and is shown as the innermost ring. The outermost circle displays the locations of AMR genes (*bla*_IMP-6_ integron, *bla*_CTX-M-2_, *tetA* and *tetR*) in pKPI-6. (**c**) Comparison of *bla*_KPC-2_ *, bla*_CTX-M-65_ and *bla*_TEM-1B_-carrying complete plasmids from MS5538 with four publicly available plasmids. The plasmid from MS5538 was used as the reference and is shown as the innermost ring. (**d**) Comparison of *bla*_CTX-M-15_-carrying complete plasmids from MS5783 and MS5784 strains with two publicly available plasmids. The plasmid from MS5783 was used as the reference and is shown as the innermost ring.

Among 21 AMR-HMV isolates, five were positive for the *rmpA* (N2531, N2576, N454 and N579) or *rmpA2* (MS5288) genes and some other virulence genes (MS5288, N2531 and N2576: yersiniabactin, colibactin, aerobactin and salmochelin; N454 and N579: aerobactin and salmochelin) (Fig. 3). The complete plasmid sequences of these five strains were obtained and used for plasmid comparison with BRIG. The results demonstrated that these isolates carried a plasmid homologous to the virulence plasmid pLVPK with ≥82 % coverage and ≥99.52 % sequence identity (Fig. 5a, Table S1, available in the online version of this article). The remaining 16 AMR-HMV isolates were negative for both *rmpA* and *rmpA2* genes (Fig. 3).

The complete plasmid nucleotide sequence comparison by BRIG confirmed that 11 isolates possessed pKPI-6-like plasmids with ≥96 % coverage and ≥99.81 % sequence identity to pKPI-6, which possessed a *bla*_IMP-6_-carrying integron and *bla*_CTX-M-2_ (Fig. 5b, Table S1). The plasmid pKPI-6 is an IncN plasmid that has caused an epidemic in West Japan due to its dissemination within the family *Enterobacteriaceae* [29].

Strikingly, 5/11 isolates carrying pKPI-6-like plasmids also carried the pLVPK-like virulence plasmid (Fig. 3), suggesting the convergence of hypervirulence and antimicrobial resistance in these clones. Among these, one ST36 isolate (MS5288) carried a plasmid with very high homology to the virulence plasmid from WCHKP13F2, an ST36 hypervirulent-carbapenem-resistant Kp reported from China [30] (100 % coverage, 99.96 % identity) (Fig. 5a). Strain WCHKP13F2 was reported to have an intact *rmpA* and truncated *rmpA2*, whereas our isolate MS5288 carried an intact *rmpA2* and a truncated *rmpA* due to the insertion of a stop codon. In terms of AMR elements, WCHKP13F2 carried a *bla*_KPC-2_-harbouring plasmid, whereas MS5288 carried a plasmid co-expressing *bla*_CTX-M-2_ and *bla*_IMP-6_.

Two isolates carried plasmids with the coexistence of *bla*_KPC-2_, *bla*_CTX-M-65_, and *bla*_TEM-1B_ (MS5538 and MS5544). The plasmid derived from MS5538 showed homology with the publicly available pKPC-HvKP4 (96 % coverage, 99.94 % identity) and p675920-1 (96 % coverage, 99.97 % identity) (Fig. 5c). Plasmid p675920-1 was reported to be a hybrid of pHN7A8 from *Escherichia coli* (carrying *bla*_CTX-M-65_ and *bla*_TEM-1B_) and pKPC-KL30 (carrying *bla*_KPC-2_) [31].

Two isolates (MS5783 and MS5784) carried *bla*_CTX-M-15_ and *bla*_TEM-1B_ on the same plasmid, with 100 % homology between the two isolates (Fig. 5d). The MDR region, which harbours *bla*_CTX-M-15_ (β-lactam resistance), *bla*_TEM-1B_ (β-lactam resistance), *sul2* (sulphonamide resistance), *aph(6)-Id* (aminoglycoside resistance), *aph(3’)-Ib* (aminoglycoside resistance), *qnrB1* (fluoroquinolone resistance), *tetA* (tetracycline resistance) and *dfrA14* (trimethoprim resistance), was partially homologous to that of pAMA1416 (44 % coverage, 100 % identity) and pCTX15_DHQP14000954 (44 % coverage, 99.99 % identity) (Fig. 5d).

### String level and temperatures

**Fig. 6.**
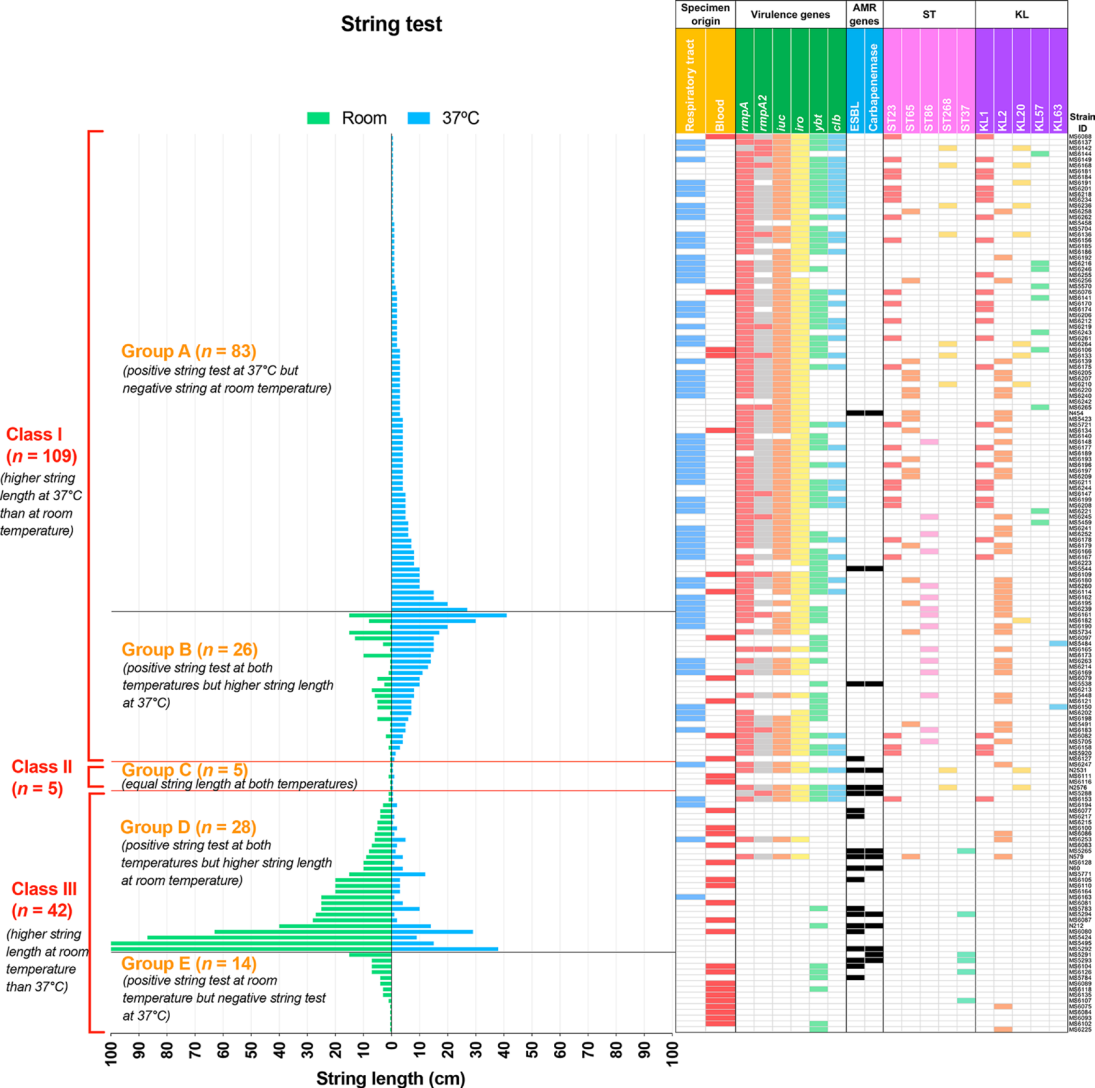
Correlation among temperature, string length and other characteristics. String lengths of each isolate at room temperature (green bars) and 37 °C (blue bars) are presented and separated into three classes: Class I, higher string length at 37 °C than at room temperature; Class II, equal string length at both 37 °C and room temperature; and Class III, higher string length at room temperature than at 37 °C. Temperature-dependent string patterns within Class I and Class III are further divided into subgroups: group A, positive string test at 37 °C but negative string test at room temperature; group B, positive string test at both temperatures but higher string length at 37 °C; group D, positive string test at both temperatures but higher string length at room temperature; and group E, positive string test at room temperature but negative string test at 37 °C. Class II comprises only one group, namely group C. The corresponding characteristics of each isolate, including specimen origin, virulence genes, AMR genes, ST and KL, are displayed in the right panel. For the *rmpA* and *rmpA2* columns, red colour indicates an intact gene, and grey colour indicates a truncated gene.

We re-ordered the isolates according to string length at 37 °C or room temperature and combined this with the other key features, such as specimen origins, virulence factors, ESBL and carbapenemase genes, ST and K-loci (Fig. 6).

According to string test categorization (see Methods), among 156 string test-positive isolates, 69.9 % (*n=*109) belonged to Class I, 3.2 % (*n=*5) belonged to Class II and 26.9 % (*n=*42) belonged to Class III. More specifically, 53.2 % (*n=*83) of isolates showed positive string tests only at 37 °C (negative string test at room temperature) (group A), 16.7 % (*n=*26) of isolates showed positive string tests at both temperatures but higher string length at 37 °C than at room temperature (group B), 3.2 % (*n=*5) of isolates produced equal string lengths at both temperatures (group C), 17.9 % (*n=*28) of isolates showed positive string tests at both temperatures but higher string length at room temperature than at 37 °C (group D), and 9.0 % (*n=*14) of isolates showed positive string tests only at room temperature (negative string tests at 37 °C) (group E).

The mean string length of Class I at 37 °C was significantly higher than that of Class III (5.67±0.63 vs 4.04±1.20 cm, *P*<0.001), while the mean string length of Class III at room temperature was significantly higher than that of Class I (17.32±3.91 vs 0.10±0.27 cm, *P*<0.001, Table 1). Within Class I, group B produced a significantly higher mean string length at 37 °C than group A (11.21±1.74 vs 3.93±0.48, *P*<0.001, Table 2).

### HMV phenotype and *rmpA*/*rmpA2* genes

**Fig. 7.**
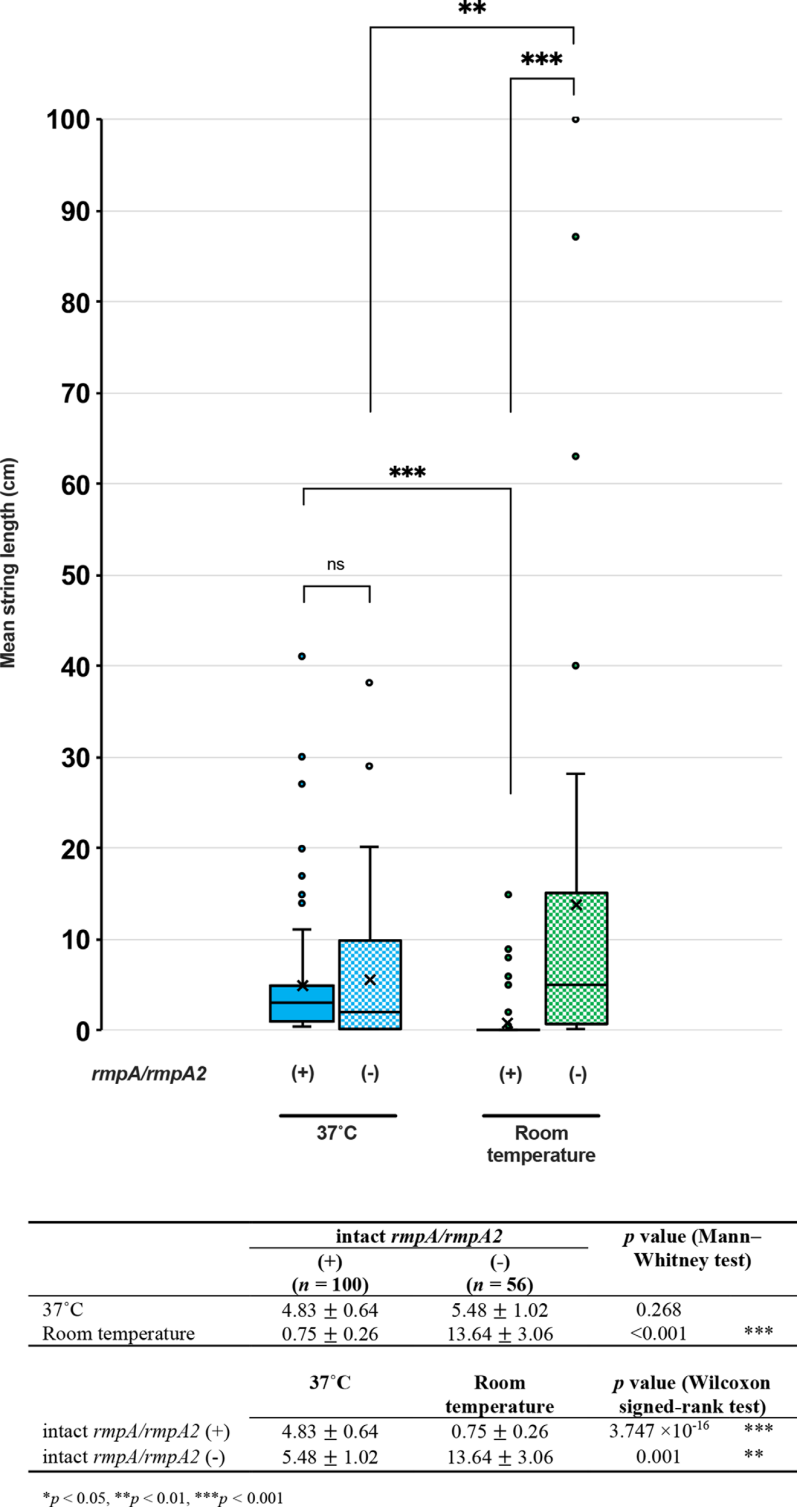
The presence of *rmpA*/*rmpA2* genes and mean string length at 37 °C and room temperature. The isolates are divided into *rmpA/rmpA2-*positive and *-*negative groups, and the mean string lengths (cm) of each group at 37 °C and room temperature are demonstrated. Comparison of the mean string length between two groups at the same temperature was analysed with a Mann–Whitney test, and comparison of the mean string length between two temperatures of the same group was analysed with a Wilcoxon signed-rank test. ns, Not significant; ***P<0.001.

We detected 101 isolates carrying the *rmpA* gene, among which three isolates (3.0%) possessed truncated *rmpA* genes (Fig. 6). Conversely, 88 isolates carried *rmpA2* genes, 73 (83.0 %) of which were truncated *rmpA2* genes. Thirteen strains (8.3%) carried both intact *rmpA* and intact *rmpA2*. Altogether, 100 isolates harboured an intact *rmpA* and/or *rmpA2* [henceforth referred to as the *rmp* (+) group], and 56 isolates carried a truncated or were negative or for both *rmpA* and *rmpA2* [henceforth referred to as the *rmp* (-) group]. A comparison of the mean string length between the *rmpA-*positive and *rmpA*-negative groups revealed no significant difference in string length at 37 °C, but the string length at room temperature in the *rmp* (-) group was significantly higher than that of the *rmp* (+) group (Mann–Whitney test, *P*<0.001) (Fig. 7). Within the *rmp* (+) group, the mean string length at 37 °C was significantly higher than that at room temperature (Wilcoxon signed-rank test, *P*<0.001), whereas, within the *rmp* (-) group, the mean string length at room temperature was significantly higher than that at 37 °C (Wilcoxon signed-rank test, *P*<0.001).

### Genomic comparisons of Class I and Class III isolates

Class I isolates were commonly distributed in specific STs, such as ST23 (*n=*26, 23.9%), ST65 (*n=*18, 16.5%), ST86 (*n=*14, 12.8%) and ST268 (*n=*6, 5.5%) (Fig. 6). Conversely, Class III isolates were highly diverse, with the most common STs being ST37 (*n=*6, 14.3%), ST45 (*n=*3, 7.1%), and certain sporadic STs such as ST147, ST17, ST34 and ST611. ST23, ST65 and ST86 were significantly more prevalent among Class I isolates, while ST37 was more so among Class III isolates (Table 1). Within Class I, ST86 was significantly more common in group B than in group A (*P*=3.738×10^−3^, Table 2).

Given the known associations between the STs and K-loci, particularly among the STs that were overrepresented in the Class I group (described above), it is unsurprising that KL2 (*n=*35, 32.1%), KL1 (*n=*28, 25.7%), KL57 (*n=*10, 9.2%) and KL20 (*n=*9, 8.3%) were most frequently found in Class I, whereas Class III isolates were associated with the more diverse K-loci. Similarly, isolates carrying *rmp* (+), which were overrepresented among the well-known hv-clones (ST23, ST65 and ST86), were significantly more prevalent among Class I isolates than Class III isolates (85.3 % vs. 9.5 %, *P=*3.104×10^−18^, Table 1, Fig. 6). This was also the case for the key acquired virulence determinants coding for yersiniabactin (*ybt*), colibactin (*clb*), aerobactin (*iuc*) and salmochelin (*iro*) (*P*<0.001, Table 1, Fig. 6). A virulence score of 0 (VS0) was significantly associated with Class III (*P*<0.001), while VS3, VS4 and VS5 were significantly associated with Class I (Table 1). Conversely, ESBLs and carbapenemases, which were rare among the hv-clones, were both significantly more common among Class III than among Class I isolates (*P*<0.001, Table 1, Figs 3 and 6).

Within Class I, the *rmp* (+) groups were significantly more common in group A than in group B (94.0 % vs. 57.7 %, *P*=4.1×10^−5^, Table 2), as were the genes for colibactin, aerobactin and salmochelin.

### Specimen origins

Among the 156 isolates, 125 with available information were analysed to investigate the characteristics associated with the specimen origins, e.g. the samples from the RT, bloodstream infection (BSI), urinary tract infection, bile or ascitic fluid. The majority of specimens were collected from the RT (*n=*67, 53.6%) and BSI (*n=*34, 27.2%), with the rest from urine (*n=*9, 7.2%), bile (*n=*5, 4.0%), ascitic fluid (*n=*3, 2.4%), pus (*n=*2, 1.6%), drainage (*n=*2, 1.6%) and other miscellaneous origins.

The isolates derived from the RT and BSI showed clear differences with regard to the STs, K-loci, *rmpA*/*rmpA2* genes, virulence genes, AMR genes and favourable/unfavourable temperature for string formation (Table 3, Fig. 3). KL2-ST65 and KL2-ST86 were significantly associated with the RT isolates (*P*<0.05, Table 3). Similarly, KL1 and KL2 were significantly associated with the RT isolates (*P*<0.05, Table 3). KL1-ST23 originated from 22.4 % of the RT isolates but only from 8.8 % of the BSI isolates; however, the difference was not statistically significant (*P*=0.092, Table 3). On the other hand, isolates from BSI were distributed in a diverse set of STs, including ST37 (*n=*2, 5.9%, Table 3) and some other uncommon STs (data not shown). Remarkably, isolates from the RT showed higher virulence than isolates from BSI; VS3 was significantly more prevalent among isolates from the RT (*P*<0.001), while VS0 was significantly more common among isolates from BSI (*P*<0.001, Table 3). In contrast, ESBL was significantly more common in the BSI isolates (11.8%, *P*=0.011). Regarding the string length under the two temperatures, Class I was significantly associated with the RT isolates (92.5 % vs 35.3 %, *P*<0.001), whereas Class III was significantly associated with the BSI isolates (58.8 % vs 6.0 %, *P*<0.001, Table 3 and Fig. 6). More precisely, group A was significantly associated with the RT origin (*P*<0.001), and groups D and E were significantly more common in the BSI isolates (*P*<0.01, Table 3). Similarly, 86.6 % of isolates from the RT possessed the *rmpA* gene, whereas only 23.5 % of isolates from BSI carried the *rmpA* gene (*P*<0.001, Table 3). Nonetheless, the key driver associated with the specimen types is unascertainable because of the strong associations among the ST, K-locus, *rmpA*/*rmpA2*, virulence genes and AMR genes.

## Discussion

This study represents the first investigation of string length distribution across different temperatures (37 °C and room temperature) and its correlation with the genomic population of Kp. We detected clear differences in the multi-locus STs, capsular serotype, string level, string test temperature*, rmpA*, virulence score, AMR factors and specimen origins by combining phenotypic and genotypic analysis of 156 HMV Kp isolates collected in Japan (shown in Fig. 4). The predominant clusters, cluster 1 (KL2-ST65), cluster 2 (KL1-ST23), cluster 3 (KL2-ST86) and cluster 4 (KL20-ST268 and KL62-ST36), shared similar characteristics, such as positive *rmpA*, *iuc* and *iro*, and the general absence of ESBL/carbapenemase (with some exceptions). These observations were consistent with the other findings among the hvKp population in Asia and Japan, especially in the case of KL1-ST23, KL2-ST65 and KL2-ST86, which have frequently been associated with severe clinical complications [3, 9, 32–34]. Among these, KL1-ST23 is well known for severe community-acquired diseases such as pyogenic liver abscesses and distinct invasive syndromes in Southeast Asia, especially in Taiwan, Hong Kong, Singapore, Korea and Vietnam [32, 35]. We found that the isolates were mostly derived from the RT and produced higher string lengths at 37 °C than at room temperature (Class I isolates). In contrast to the stereotypical hv-clones, a proportion of the isolates, especially clusters 5, 6, 7 and 8 (ST37, ST11, ST45 and ST147), displayed distinct characteristics such as the absence of *rmpA*, *iuc* and *iro* and the presence of ESBL and/or carbapenemases. Strikingly, these isolates were frequently derived from BSI and produced higher string lengths at room temperature than at 37 °C (Class III isolates).

When investigating the molecular mechanism of HMV, we observed an interesting correlation between mean string length and the presence of *rmpA* (Figs. 6 and 7). The strains carrying *rmpA* produced short strings at 37 °C, while they were nearly negative at room temperature. In contrast, the strains lacking *rmpA* could produce a certain amount of HMV at 37 °C, similar to the *rmpA* (+) isolates, but produced significantly higher HMV at room temperature. RmpA may be responsible for HMV mainly at 37 °C, which can produce relatively short strings, while a novel factor may be responsible for the HMV phenoype at room temperature, which can produce considerably high string lengths. However, the strong associations among the ST, K-locus, *rmpA*, virulence genes and AMR genes make it difficult to specify the single genetic factor that drives the association with different HMV patterns and specimen origins. Nonetheless, we hypothesize that Class III isolates are more likely to stick to the instruments because of their high HMV at room temperature, which may enhance their propagation and eventually result in nosocomial outbreaks of AMR Kp in hospital settings. Meanwhile, Class I isolates are likely to stick to the alveolar epithelial cells and pulmonary endothelial cells because of their HMV production at 37 °C, providing them with resistance against excretion from the human body.

To the best of our knowledge, no study has investigated the temperature-dependent HMV phenotype or mechanism of temperature-dependent capsule expression of Kp. The HMV phenotype is important for virulence inside the human body (37 °C), but its significance at room temperature, which may facilitate their long endurance and propagation on the surfaces and in the hospital environment (e.g. on catheters, medical equipment, water sinks, tables and chairs) remains unknown. From our observations, in most strains that lacked *rmpA* and produced high HMV at room temperature, string length could either reach its peak after 1 or 2 days or be maintained at a high level over 3 days (unpublished data), suggesting their high survival capacity and endurance under unfavourable conditions outside the human body. Furthermore, approximately two-thirds of these clusters were able to produce HMV at 37 °C (group D), implying their ability to invade the human immunity defence similar to that of the *rmp-*positive clone. There were some rare phenomena in previous reports that liver abscesses are not significantly associated with HMV or that metastasis infection was developed from ‘classical Kp’ (non-hv-Kp) [9]. Previous studies evaluated string tests by stretching a single [2, 14] or multiple colonies [7] using an inoculation loop [2, 7, 14] or a toothpick [36]. These methods provide a qualitative measurement of HMV with ‘positive’ or ‘negative’ results; however, comparative evaluation between strains with ‘positive’ string tests is difficult because of the short string length generated by only one or a few colonies. Therefore, to make the string test quantifiable, we used a cotton swab to collect an area of bacterial colonies (one-quarter of a standard agar plate) and stretched upwards so that longer strings could be generated to make the string length measurement easier. Additionally, based on our findings, we suggest a definition of HMV that is not only limited to the result of the string test at 37 °C but also at room temperature, and the novel factor responsible for HMV in the absence of *rmpA*, if discovered, should be considered as a biomarker for defining a strain as hvKp or cKp. A different study is being conducted to discover novel factors responsible for the HMV phenotype at room temperature within these clusters.

Carbapenem antibiotics have been the last resort against MDR Kp; however, carbapenemase-producing Kp has been continuously reported over the past decade. Our study detected 13.5 % (21/156 isolates) of the HMV Kp carrying ESBL/carbapenemase genes, among which 23.8 % (5/21 strains) displayed the convergence of hypervirulence and carbapenem resistance by the acquisition of pLVPK-like and pKPI-6 plasmids. These five isolates belonged to three sequence types and three capsular serotypes [ST36-KL62 (one isolate), ST65-KL2 (two isolates) and ST268-KL20 (two isolates)] (Figs 2 and 3). The pLVPK plasmid has been broadly disseminated worldwide [37], while pKPI-6 is an epidemic AMR plasmid in Japan [29]. In Japan, in addition to the pKPI-6-like plasmid-carrying ST23-hv-Kp, which was reported in 2019 [38], our study revealed that the acquisition of this AMR plasmid also occurred in ST65-hv-Kp, posing a critical threat to the extended emergence of carbapenem resistance in hvKp clones. While most carbapenem-resistant hvKp strains in China and some other countries harboured *bla*_KPC-2_ [37, 39], all five carbapenem-resistant hvKp in this study carried *bla*_IMP-6_ and *bla*_CTX-M-2_ in pKPI-6-like plasmids, suggesting a distinct epidemiological feature of Japanese clones compared with the clones from other countries. In addition, we detected six classical Kp carrying pKPI-6 like plasmids (N212, MS5292, N60, MS5293, MS5294 and MS5265) and two ST11 isolates carrying *bla*_KPC-2_ (MS5538 and MS5544), although none of them carried a pLVPK-like plasmid or virulence genes such as *rmpA*/*rmpA2*, aerobactin-, and salmochelin-encoding genes (Fig. 3).

A limitation of our study is the inability to access patient clinical data, hindering the interpretation of the association between infection with different clonal Kp and clinical outcomes. Additionally, the isolates in our study were mainly collected from West Japan, which may not precisely reflect the HMV Kp population in Japan. Future surveillance will be carried out to investigate the characteristics of HMV Kp from all over Japan, as well as to compare the geographical epidemiology among different areas. Another limitation is that the results of the modified string test may not be entirely compatible with those of the conventional string test. In this study, we performed the string test at two different temperatures and three different time points, making it complex and time-consuming, and unsuitable for screening in clinical laboratories. Nevertheless, we standardized the string test protocol to make it reliable for evaluating the temperature-dependent HMV phenotype, which may contribute to the future exploration of the HMV regulatory mechanism under different conditions.

In summary, our analysis of HMV Kp showed that the low HMV level is usually related to *rmpA*/*rmpA2*, while the high HMV level is not. Strains carrying *rmpA*/*rmpA2* are likely to express HMV at 37 °C, whereas those negative for these genes are likely to express HMV at room temperature. On the one hand, some clusters shared similar characteristics, such as positivity for *rmpA*, mostly without ESBLs/carbapenemases, mostly derived from the RT, and produced higher string lengths at 37 °C than at room temperature. Some clusters, however, showed distinct characteristics such as testing negative for *rmpA*, positive for ESBL and/or carbapenemases, frequently derived from BSI, and produced a higher string length at room temperature than at 37 °C. Our findings may facilitate the future discovery of unknown factor(s) responsible for HMV at room temperature and may explain different virulence tactics of Kp infection.

## Supplementary Data

Supplementary material 1
