# Supplementary material for: Genomic epidemiology and temperature dependency of hypermucoviscous Klebsiella pneumoniae in Japan
Source: Microb Genom. 2022 May 27;8(5):mgen000827. doi: 10.1099/mgen.0.000827 (PMC9465067; doi:10.1099/mgen.0.000827)
Supplement: Supplementary material 1 [file mgen-8-827-s001.pdf]

Supplementary Material

Supplementary Table

Table S1. Comparison between pLVPK or pKPI-6 with homologous plasmids derived from our isolates

Virulence plasmid derived from	Size (bp)	Compare to pLVPK (219,385 bp)	
		Coverage (%)	Identity (%)
MS5288	207,201	92	99.58
N454	224,703	95	99.62
N579	226,407	97	99.59
N2531	189,954	82	99.94
N2476	203,229	87	99.52

pKPI-6-like plasmid derived from	Size (bp)	Compare to pKPI-6 (52,413 bp)	
		Coverage (%)	Identity (%)
MS5288	51,966	100	99.88
N2531	52,450	100	99.81
N2576	52,450	100	99.81
N454	49,602	96	100
N579	52,196	99	99.93
N212	49,602	96	99.99
MS5292	52,413	100	99.83
N60	51,064	97	100
MS5293	52,063	99	99.87
MS5294	52,413	100	100
MS5265	50,839	97	100
MS5291	20,408	39	99.87
